# Supporting children’s numeracy competencies and families’ HNE: Exploring the role of apps and digital parent information in STEM vs. Non-STEM families

**DOI:** 10.1007/s10212-025-00953-7

**Published:** 2025-03-21

**Authors:** Anna Mues, Efsun Birtwistle, Astrid Wirth, Tina Schiele, Frank Niklas

**Affiliations:** 1 Department of Psychology, Ludwig-Maximilians-Universität München, Munich, Germany; 2 School of Psychology, University of Nottingham, Nottingham, UK; 3 Department of Education, University of Vienna, Vienna, Austria

**Keywords:** Family Intervention, Home Numeracy Environment, Numeracy Competency Development, App-based-Learning, STEM, Learning4Kids

## Abstract

Early numeracy competencies are of great importance for children’s competency development. Here, early digital intervention approaches offer the potential to support all children and their families. We investigated whether the provision of specific numeracy learning apps and parent information about children’s numeracy development improves both, children’s numeracy competencies and the quality of families’ home numeracy environment (HNE) while considering the potential impact of parental (STEM) occupation. Children’s numeracy competencies were measured twice in two cohorts (*N*_*1*_ = 190 children; *M*_1age_ = 63.6 months; *SD*_*1*_ = 4.4; *N*_*2*_ = 310 children; *M*_2age_ = 59.4 months; *SD*_*2*_ = 3.9) with a six-month interval between t1 and t2. Parents were surveyed about the family characteristics and the HNE. Families in the numeracy intervention group (*N*_total_ = 151) received tablet computers with specific numeracy learning apps and parent information. No significant intervention effect on the quality of the HNE was found. However, children from intervention families showed significantly greater numeracy competency gains, even when considering child and family characteristics and independent of parental occupations. Consequently, high-quality learning apps can support the development of children’s numeracy competencies already at preschool age. Further ideas on how to reach families and enhance the HNE are discussed.

**Supplementary Information:**

The online version contains supplementary material available at 10.1007/s10212-025-00953-7.

## Introduction

Children’s early numeracy competencies are key predictors for later mathematical achievement and success (Duncan et al., 2007; Niklas & Schneider, 2017) and vary greatly by the time they start school (Dowker, 2008; Gould, 2012). As these differences tend to persist until later school years (Davis-Kean et al., 2022), there is a need for high-quality learning opportunities from a very young age onwards.

In response to this demand, there has been an increasing focus on mathematics education and intervention approaches to support the development of young children’s mathematical competencies worldwide (e.g., Niklas et al., 2020). As children grow up in media-rich homes and are in contact with digital tools in daily life, digital interventions that use mobile technology such as tablet computers (tablets) seem to be a promising approach in supporting children’s mathematical competencies (Niklas et al., 2020, 2022). Indeed, intervention studies showed that tablets can act as an encouraging educational tool to enhance children’s and families’ learning environments (Niklas et al., 2021). Although, intervention studies indicate that the use of learning applications (apps) may appeal to all children and families and seem to have a positive impact on children’s numeracy learning (Berkowitz et al., 2015; Kim et al., 2021; Papadakis et al., 2018), more research is needed on the effectiveness of tablet based-approaches, especially large-scale randomized trials with a focus on children’s development (Griffith et al., 2020).

Additionally, the family and the home numeracy environment (HNE) (Niklas & Schneider, 2014) as well as family characteristics, such as parental occupation (Omolade et al., 2014) and more specifically parents’ occupation in a field related to Science, Technology, Engineering and Mathematics (STEM), were shown to be important predictors of children’s mathematical development (Mues et al., 2021; see Plasman et al., 2021 for an overview). The HNE provided by parents with STEM occupations is characterized by more frequent and higher-quality everyday mathematical interactions and activities. Such interactions have proven to be particularly supportive of children’s mathematical development (Zippert & Rittle-Johnson, 2020).

However, not all families seem to be able to support their children’s development in an adequate way. For instance, disparities in socioeconomic status contribute to differences in the quantity and quality of HNE activities in a family with high SES families providing more and higher quality HNE activities compared to families with lower SES, who often provide HNE activities less often and in a lower quality (Anders et al., 2023; Eason et al., 2022; Niklas & Schneider, 2014).

Even though digital interventions offer a non-intensive way to support families (Lehrl et al., 2021; Nelson et al., 2024), there are currently only a few studies that have investigated the effectiveness of supporting the HNE with digital interventions on the one hand and their independence from family background on the other hand, indicating a clear research gap (Alam & Dubé, 2023a; Cohrssen et al., 2023; Griffith & Arnold, 2018).

Here, the question arises of how best to support children’s development of numeracy competencies and the quality of the HNE independently of family background and resources when using apps and digital parent information on children’s early numeracy development. Consequently, we developed a tablet-based intervention approach to support children’s numeracy competencies by using learning apps and evaluated whether this approach was successful and whether potential intervention effects were associated with parental STEM occupations.

## Children’s early numeracy learning and family characteristics at home

Early numeracy competencies include a variety of number-based skills (Krajewski & Schneider, 2009) and were shown to be strong predictors of later mathematical performance (Devlin et al., 2022). Here, important steps for the development of quantity-number competencies and the transition from a procedural to a conceptual understanding of numerals and arithmetic skills are the identification of basic numerals, the recognition and understanding of the linkage between numbers and words as well as the ability to count, compare, order and discriminate quantities (e.g., Geary & van Marle, 2018; Schneider et al., 2017). When examining children’s development of numeracy competencies, further precursors such as cognitive abilities (e.g., intelligence) and other child characteristics (e.g., age and sex) should be considered (Schneider et al., 2014).

In addition, the HNE children grow up in is a strong predictor of children’s early numeracy competencies (Mutaf-Yıldız et al., 2020; Niklas & Schneider, 2014; Susperreguy et al., 2020a). The HNE can be differentiated in two constructs: the informal HNE, which consists of family interactions and activities such as reading a number-related book and playing a dice game, and the formal HNE, which includes, for example, direct numeracy-related instructions and teaching to support children’s numeracy learning in the home environment (LeFevre et al., 2009; Niklas & Schneider, 2014). Consequently, interventions aimed at improving early numeracy competencies may target families’ HNE (Nelson et al., 2024). Here, a meta-analysis by Nelson et al. (2024) emphasized that intervention approaches that included parental information and regular provision of materials and support for caregivers had the strongest effects on the families’ HNE.

Furthermore, parental occupations, and here especially STEM occupations, were shown to be an important predictor for students’ mathematical achievement (OECD, 2014) and children’s numeracy competencies in early years (Mues et al., 2021). For instance, Mues et al. (2021) showed that parents’ learned STEM occupation was directly positively associated with children’s numeracy outcomes and correlated with the families’ HNE. Here, the authors expect parents whose occupational background was related to STEM to engage in more frequent and higher-quality mathematical interactions and activities with their children at home. Therefore, parents with an occupational background with no relation to STEM may need more guidance and information about high-quality mathematical interactions and activities with their children.

However, not only the frequency of analog mathematical activities and interactions in the family as well as family characteristics (Eason et al., 2022; Elliott & Bachman, 2018) are associated with children’s early numeracy development, but also the usage of appropriate digital tools and apps (e.g., Lehrl et al., 2021; Moyer-Packenham et al., 2016; Papadakis et al., 2018).

## Digital technology in contexts of children’s numeracy learning

Multi-touch technology availability and its usage have increased over the last decade. Therefore, children have become major consumers of these devices (Papadakis et al., 2018). Digital media enables the support of children’s mathematical development and opens up the possibility of addressing traditional issues in the field of education such as accessibility to education for all children and their families (Papadakis et al., 2018). Children’s performance in numeracy tasks can be supported by digital tools such as tablets with apps (e.g., Berkowitz et al., 2015), also for children who lack experience with digital devices (Lee & Choi, 2020).

Special features of tablet technologies, in comparison to traditional media, are interactivity, easy usage, accessibility, and the possibility of accurate and digital measurement (Semmelmann et al., 2016) for example, by using mobile sensing technology (Birtwistle et al., 2022) to collect valid app usage data in interventions or experiments. The use of tablets as a developmentally appropriate approach has been proven to support children’s learning and development effectively (Papadakis et al., 2018) in comparison to a typical (analog) learning situation (e.g., classroom, Calder, 2015). Furthermore, the usage of learning apps in the early childhood context, for example in kindergartens, primary schools, and the home learning environment, has increased (see Hirsh-Pasek et al., 2015). Learning apps have successfully been used to improve numeracy learning (Outhwaite et al., 2019; Papadakis et al., 2021), not only in laboratory settings but also in naturalistic contexts such as the family environment (e.g., Berkowitz et al., 2015; Niklas et al., 2024; Papadakis et al., 2018). However, the usage of educational learning apps does not necessarily imply appropriate, supportive, and meaningful content and support for children (see Hirsh-Pasek et al., 2015). Therefore, several theoretical evaluation frameworks have been developed to summarize what an educational app should include (Hirsh-Pasek et al., 2015; Kolak et al., 2021; Papadakis et al., 2020). For instance, Hirsh-Pasek et al. (2015) suggested that children learn best when they are cognitively engaged and active, when they experience the app as meaningful as well as socially interactive, and when the app is guided by a specific educational goal. However, Meyer et al. (2021) have already shown that the majority of apps available in public app stores do not fulfil the requirements formulated by Hirsh-Pasek and colleagues. And even though many evaluation tools have been developed over the past years, there is still no consensus on the criteria for a high-quality learning app (Kim et al., 2021; Papadakis, 2021).

Tablets further offer the chance to enhance the quality of the (home) learning environments (Lee & Choi, 2020; Lehrl et al., 2021; Papadakis et al., 2018) and parent–child interactions (Zippert et al., 2019).

## Digital interventions at home

Compared to research on the home literacy environment, research on the digital support of the home numeracy environment is scarce. However, there are some findings about the support of parent–child interactions and math talk while using apps (see Niklas et al., 2021 for an overview). For instance, Berkowitz et al. (2015) reported a significant increase in mathematical achievement by the end of the school year when children frequently used a math story app with their parents about once a week. These math-related interactions were also benefiting children whose parents were anxious about doing mathematics. Further, Zippert et al. (2019) reported an increase in parent–child interactions while playing with an app-based digital board game. Parents who had received additional information on how to interact with their children with mathematics focus produced more math-related talk compared to parents and their children who only played the digital math board game without any further instructions or information. Similarly, Griffith and Arnold (2018) pointed out that both children and parents were engaged when interacting with apps: however, they had different roles. Children interacted in a direct way with the app, whereas parents’ main role was to help and contribute to the interaction verbally. In contrast, when interacting with analog tools such as a book or a math toy, parents showed warmer and more playful behavior and interactions. The authors assumed that this difference between digital and non-digital tools may reflect a decreased comfort with apps by the parents, and that not much of a leading role is needed when using apps.

Mobile phones were utilized in a study by Cohrssen et al. (2023) as an easily accessible tool of intervention that enabled caregivers to support their children’s learning at home. Four short videos were provided to the parents through the messenger app WhatsApp, with a focus on the importance of the home learning environment, of playing with blocks and of counting games, and on how to encourage dialogic reading. In addition, building blocks or a story book were sent to families’ homes. No significant change in the ‘mathematics importance’ scale was found for the intervention group and no significant effects were demonstrated when comparing the ‘measurement’ and ‘counting’ scales over time for the intervention and the control group. However, significant increases in mean scores for the ‘measurement’ and ‘counting’ scales were reported within the intervention group.

These studies show divergent findings on how to support families’ activities and interactions at home with digital intervention approaches. However, while studies on analog settings reported specific family characteristics such as SES or parents’ STEM occupation as influencing factors for children’s numeracy development and families’ HNE (Mues et al., 2021), there are no comparable studies on digital settings so far.

## The present study

With our intervention approach, we aimed to support children’s numeracy competencies using researcher-developed learning apps for tablets and to enhance the HNE by providing parents with information on children’s early numeracy development, while also considering potential differences based on parents’ STEM occupations. Consequently, we examined the following research questions and put forward the following hypotheses:

**RQ 1** Does a digital intervention with learning apps improve pre-schoolers’ numeracy competencies and is this improvement associated with parents’ STEM occupation?

**H 1.1** High-quality learning apps improve preschoolers’ numeracy competencies over the time of the intervention period.

**H 1.2** Children’s improvement through the intervention is associated with their parents’ STEM occupations, with higher numeracy outcomes for children whose parents have an occupational STEM background in comparison to children whose parents do not have an occupational STEM background.

**RQ 2** How frequently did parents use the provided information (guidelines and tips), and are the reported usage times, the actual usage times, the HNE, and the parents’ occupational STEM background associated with each other? As we are not aware of prior research on these questions, we used an exploratory approach and did not pose any specific hypotheses.

**RQ 3** Does the provided parent information on early numeracy development enhance the quality of the HNE in the families and is this also associated with parents’ STEM occupation?

**H 3.1** Digital parent information on children’s early numeracy development enhances the quality of families’ HNE.

**H 3.2** Parents without a occupational STEM background benefit more from the parent information compared to parents with a occupational STEM background.

## Materials and methods

### Sample

The data used in our analysis were taken from the first and second measurement point of the EU-funded five-year longitudinal randomized field study “Learning4Kids” in Germany (Niklas et al., 2020, 2022). Children’s numeracy competencies were assessed in a total sample of *N* = 500 children from two cohorts with an average age of *M*_*total_age*_ = 60.96 months (*SD* = 4.61) and *n* = 257 girls and *n* = 243 boys. Cohort 1 consisted of *n*_*1*_ = 190 children with an average age of *M*_*1age*_ = 63.60 months (*SD*_*1*_ = 4.40) at the first measurement point. Cohort 2 included *n*_*2*_ = 310 children with an average age of *M*_*2age*_ = 59.36 months (*SD*_*2*_ = 3.94). From the first to the second measurement point, there was a drop out of a total of five families, which led to a final analytic sample of *N* = 495. Here, no significant differences between the dropped-out families were found for the study variables (*p* > 0.05). In addition to children’s assessments, parents were surveyed regarding formal and informal aspects of the HNE and family and child characteristics. For both cohorts, mostly mothers (C1 = 72.30%; C2 = 73.90%) filled in our parent survey.

### Procedure

Cohort 1 families were mainly recruited from kindergartens and via a professional online company for study recruitment. For cohort 2, the recruiting approach was changed due to the Covid-19 pandemic and limited access to kindergartens. Therefore, 6000 families were contacted via mail with the support of the Government Department administration office in Munich, which provided contact details of families with children in the targeted age group. The families received a description of the study, an invitation to participate in our project and to contact the project team via e-mail or telephone. Afterwards, all families who indicated their interest in participating were contacted and written formal consent was collected during the first family visit. The European Research Council Executive Agency and the Ethics committee of the Faculty of Psychology and Educational Sciences at the University of Munich approved all research activities.

In cohort 1, *n*_*1*_ = 65 and in cohort 2, *n*_*2*_ = 91 children were randomly assigned to the numeracy group. Families received a tablet computer with specific numeracy learning apps and parent information on numeracy development and support. The control group included children without a tablet and children who received a tablet with content supporting other domains such as early literacy or cognitive development in general. The first family visit and child assessment were conducted before and after the tablet-intervention phase of 168 days at home, except for a few families whose decision was to visit the university for the assessments. The standardized assessments of children’s numeracy competencies lasted around 30–40 min. At the end of the visit, each child received a small gift. Cohort 1 was first visited in summer 2020 and reassessed in winter 2021, whereas the first assessment of cohort 2 took place in February and March 2021.

### Intervention

The intervention of the “Learning4Kids” study focuses on a tablet-based approach in the home context. After the first family visit and child assessment, families received a tablet containing the learning apps of the study for usage at home during the intervention period. Therefore, 18 new learning apps were developed and designed by researchers, students and an app-developer (see Niklas et al., 2020; for more detail see Supplement 1). All developed numeracy apps aimed to support efficient numeracy learning and followed the framework of Hirsh-Pasek et al. (2015), who suggested that children learn best when they are cognitively engaged and active, when they experience an app as meaningful as well as socially interactive, and when they are also guided by a specific educational goal. The apps focused mainly on topics in the context of numeracy activities, for example counting, number drawing and sorting, number learning, learning the clock, and measurements. Different forms of numerical values such as Arabic numbers, dots, dice or fingers were used. Furthermore, numbers tapped on during app usage were always verbalized. Consequently, further competencies, apart from number recognition learning, were implemented in most of the apps.

Children started the intervention with five learning apps focusing on basic numerical content and received additional learning apps each month with a total of 18 apps available in the fifth and final month (see Niklas et al., 2020). The difficulty level of the apps started from very easy and gradually increased to more difficult and challenging levels (e.g., higher numbers, more distractors, more complex tasks).

To support not only children’s numeracy development directly but also their families’ HNE, parent information was provided for the parents on the tablet computer via a PDF reader (MuPDF reader). The parent information was provided in form of guidelines which included topics such as mathematics and numbers in everyday life and parents as role models (see Niklas et al., 2020; for more detail see Wirth et al., 2022; for an overview of the topics, see Supplement 2 Table 1 and Example 1). New information was automatically installed on the tablet every month. Additionally, parents received weekly tips on how to engage in mathematics together with their children at home (see Supplement 2 Table 1 and Example 2).

Aligning with recommendations by Gearing et al. (2011), before starting the assessments and first intervention phase, a comprehensive assessment training for all team members and research assistants as well as detailed intervention protocols were provided. During the first family visit, parents received written guidelines and verbal instructions on how to use the devices and where to find support, if needed. A recommendation of 20 min usage time per day was given. However, this was not mandatory, and the families were instructed to decide about the use of the tablet in their everyday life context according to their and the child’s individual preferences. The usage times of all apps and parent information between t1 and t2 were monitored via an app on the tablet (‘Phone study app’, PSA), using mobile sensing (Birtwistle et al., 2022; Niklas et al., 2022). The PSA was also used as a reward system for the children. A new reward (a virtual animal sticker on a world map) appeared after every 30 min of app usage with a maximum of two stickers achievable within 24 h.

### Measures

#### Children’s numeracy competencies

To assess children’s numeracy competencies, different standardized tests were used. The MARKO-Screening test (“mathematics and concepts of calculation before school entry”, MARKO-S; Ehlert et al., 2020) consists of 21 items concerning cardinality, numbers, number division, ordinal number bars, as well as inclusion and relations (McDonald’s ώ t1 = 0.80; t2 = 0.79). Also, an adapted version of a standardized calculation subtest with eight items (“Assessment of basic mathematical competencies in kindergarten”; Krajewski, 2018) was used. Here, children’s addition and subtraction skills were tested (McDonald’s ώ t1 = 0.76; t2 = 0.74). Moreover, several subtests of the “Würzburger preschool test: Assessments of literacy and mathematical (precursor) abilities and linguistic competencies in the last year of kindergarten” were applied (Endlich et al., 2017): number sequences forward, number sequences backward, knowledge of numerical representations and number symbol knowledge. Each subtest comprises eight items with the exception of number representation with ten items (McDonald’s ώ t1 = 0.93; t2 = 0.94). A sum score of all subtests was used in the analysis (McDonald’s ώ t1 = 0.94; t2 = 0.95).

#### Home numeracy environment

Parents were asked to report on the formal and informal HNE (LeFevre et al., 2009) with five (McDonald’s ώ t1 = 0.77; t2 = 0.76) and ten items (McDonald’s ώ t1 = 0.69; t2 = 0.68; adapted from Niklas et al., 2016). Here, statements in the context of teaching mathematical concepts (e.g., “At home, I specifically show my child numbers and how to write them”) or questions about the engagement in everyday numeracy activities such as “How often do you involve your child in weighing and counting food and paying at the counter when you go shopping?” were rated on a five-point Likert scale (e.g., “does not apply at all” to “does exactly apply”, or “several times a week” to “never”). Values of 4 to 0 were assigned accordingly, with higher values indicating a higher-quality HNE. The mean of both, formal and informal items, was calculated and used to measure a global HNE construct (McDonald’s ώ HNE_total_ t1 = 0.84; McDonald’s ώ HNE_total_ t2 = 0.77).

#### Family and child characteristics

Further, parents were questioned about their learned occupation, which were categorized into occupations with and without a STEM background, as education and occupation are considered as strong predictors of SES (OECD, 2014; Omolade et al., 2014). In particular, a study conducted by Mues et al. (2021) demonstrated that parents’ learned STEM occupation is associated with children’s numeracy competencies and families’ HNE. We thus decided to take this variable as a measure of SES into account.

The categorization of the STEM (e.g., biologist, IT-specialist, geotechnical engineer, surveying technician) vs. Non-STEM background (e.g., insurance employee, preschool teacher, sales person) was based on job classifications by the German Job Agency (Bundesagentur für Arbeit, 2013, 2017). Two independent researchers coded the occupations of all parents as 1 = STEM and 0 = Non-STEM. They agreed almost perfectly according to the thresholds of Kappa reported by Landis and Koch (1977) with an intercoder reliability of Cohen’s K = 0.99 for mothers’ learned occupations and Cohen’s K = 0.98 for fathers’ learned occupations. Child characteristics such as age and sex were reported by the parents and used in our analysis as control variables. Further, children’s intelligence was also assessed as a control variable with the Columbia Mental Maturity Scale (CMMS). The scale scores range from a minimum score of 0 to a maximum score of 57 points. The split-half reliability in German contexts is between 0.92 and 0.96 (Esser, 2002).

### Usage of parent information

Parents’ usage of the provided parental information was monitored in two ways. First, the actual usage data were collected via the PSA when using the PDF reader. Second, parents reported their usage of the guidelines and tips on a four-point Likert scale (4 = “several times” to 0 = “not at all”). Further, parents were asked to rate the guidelines and tips on a seven-point Likert scale according to the German grading system (grades from 1 = excellent to 6 = insufficient and “I don’t know/ has not been read”).

## Results

Data analyses were performed with IBM SPSS Statistics 28.0 (IBM Corp, 2021). To examine our research questions, correlational analysis, repeated measurement analysis of covariance (repeated-measure ANCOVA) as well as stepwise linear regression analysis were conducted.

### Correlational Analysis

Table 1 provides an overview of the correlations between children’s numeracy competencies (t1 & t2), families’ HNE (t1 & t2), as well as children’s characteristics. Children’s numeracy competencies were significantly associated with families’ HNE at t1 and t2. Further, the results show a positive association between parental STEM occupations and children’s numeracy competencies as well as families’ HNE. Consequently, children whose parents had a STEM background were better in numeracy tasks and experienced a better HNE quality compared to children with parents without a STEM background. Additionally, older compared to younger children had higher numeracy as well as higher intelligence scores. Further, boys in comparison to girls scored significantly better in the numeracy tests at the second measurement point. Children’s intelligence was positively associated with their numeracy competencies and boys showed lower intelligence scores than girls at t1.

**Table 1:** Correlational analysis for children’s numeracy competencies, families’ HNE, parental STEM occupation, and child characteristics

	2	3	4	5	6	7	8
NumC1 (1)	0.857^**^	0.222^**^	0.211^**^	0.104^*^	0.262^**^	0.242^**^	−0.072
NumC2(2)	1	0.220^**^	0.228^**^	0.120^**^	0.276^**^	0.265^**^	−0.093^*^
HNE1 (3)		1	0.643^**^	0.169^**^	−0.054	0.095^*^	−0.030
HNE2 (4)			1	0.093^*^	−0.016	0.060	0.003
STEM^a^ (5)				1	−0.078	0.058	0.082
Age (6)					1	0.150^**^	−0.053
Intelligence (7)						1	0.126^**^
Sex^b^ (8)							1

*Note.* Pearson’s r correlation coefficients; *N* = 491–500. NumC = numeracy competencies, HNE = home numeracy environment, STEM = parental STEM occupation, 1 = t1, 2 = t2. ^a^ Non-STEM = 0, STEM = 1, ^b^ female = 1, male = 0. * *p* < 0.05; ** *p* < 0.01

### Intervention effect of the tablet-based intervention on children’s numeracy competencies development

We found a significant intervention effect of our tablet intervention on children’s numeracy outcomes (*F* (1,490) = 15.04, *p* < 0.001, *η*^*2*^ = 0.03) after considering children’s age, sex and intelligence. Children who received the numeracy tablet intervention showed a significantly greater numeracy gain over time in comparison to children who did not use numeracy learning apps.

### Association of the development of children’s numeracy competencies through the tablet-based intervention with parents’ STEM occupation

**Fig. 1:**
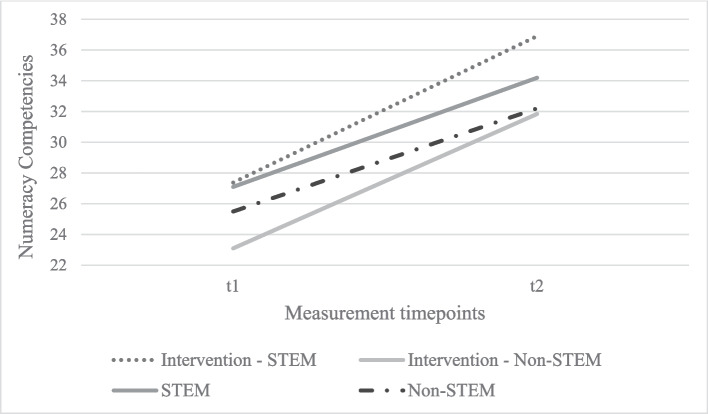
Intervention effect of the tablet-based intervention on children’s numeracy competencies and its association with parents’ STEM occupation

Further, no significant effect on the development of numeracy competencies was found for parents’ STEM occupation (*F* (1,479) = 1.03, *p* = 0.31, *η*^*2*^ = 0.002). Parents’ STEM occupation did not interact significantly with the digital intervention (see Fig. 1). However, between-subject effects show that children whose parents have a STEM occupation performed better in numeracy across t1 and t2 (*F* (1,479) = 11.153, *p* < 0.001, *η*^*2*^ = 0.023).

### Parents’ usage of digital parent information, the HNE and parents’ STEM occupation

To have a more detailed look into parents’ usage of parental guidelines and tips, the actual as well as the reported usage times and parents’ ratings were considered in a correlational analysis (see Table 2). A positive correlation between the reported usage of parental guidelines and the actual usage times was found, indicating that the reported usage time was positively associated with the actual usage times of the parents. However, no significant associations were found for the reported usage of the tips and their actual usage times.

**Table 2:** Correlational analysis for parents’ usage and rating of parental guidelines and tips with actual usage times of MuPDF Reader, the HNE, and parents’ STEM background

	2	3	4	5	6	7
HNE2^a^ (1)	0.093^*^	0.188^*^	0.210^*^	0.111	0.137	0.067
STEM^b^ (2)	1	0.065	0.064	−0.030	−0.011	0.093^*^
Guidelines_U (3)		1	0.720^**^	0.718^**^	0.643^**^	0.263^**^
Guidelines_R (4)			1	0.548^**^	0.668^**^	0.126
Tip_U (5)				1	0.801^**^	0.152
Tip_R (6)					1	0.109
MuPDF_UT (7)						1

^a^HNE2 = HNE at t2, ^b^Non-STEM = 0, STEM = 1, STEM = parental STEM occupation, Guidelines_U = parental guidelines reported usage, Guidelines_R = parental guidelines reported rating, Tip_U = parental tips reported usage, Tip_R = parental tips reported rating, MuPDF_UT = PDF reader actual usage times

Further, the more frequently parents reported using the parental guidelines, the more positive they rated them. Similarly, a longer usage time of the parental guidelines was significantly positively correlated with greater reported usage of the tips and a higher evaluation of them. Additionally, the family’s quality of the HNE at t2 correlated significantly positive with parents’ self-reported usage and ratings of the parental guidelines. This did neither apply for the tips nor for their actual usage times. Further, no significant associations were found between parents’ STEM occupation, the reported usage and the evaluation of the guidelines and tips. However, the actual usage times were significantly positive associated with parents’ STEM occupation.

### Intervention effect of the tablet-based intervention on the quality of the HNE

**Fig. 2:**
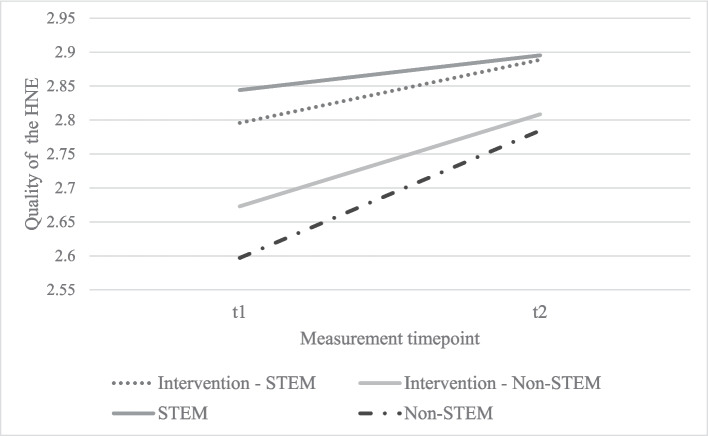
Intervention effect of the tablet-based intervention on families’ quality of the HNE and its association with parents’ STEM occupation

A repeated measurement ANCOVA was conducted to analyze the change in the quality of the HNE across groups (intervention vs. control group). No significant intervention effect (*F* (1,470) = 0.32, *p* = 0.86, *η*^*2*^ = 0.000) and effect of parents’ STEM occupation on the change in families’ quality of the HNE was found (*F* (1,470) = 2.75, *p* = 0.10, *η*^*2*^ = 0.006) (see Fig. 2). In addition, we examined the extent to which an intervention effect on the quality of the HNE might only be evident for families who received the parent information. For this purpose, we formed two subsamples with parents who used our parent information and parents who did not use the provided material at all according to their actual usage times. Here, the repeated measurement ANCOVA also showed no intervention effect (*F* (1,159) = 0.12, *p* = 0.91, *η*^*2*^ = 0.000). Finally, we calculated a stepwise linear regression analysis with the actual usage data of the MuPDF reader to predict the HNE at t2 (see Table 3). First, we included families’ HNE at t1. In a second step, we included child and family characteristics as control variables and in the last step, we accounted for the actual usage times of the MuPDF reader. The regression analysis shows that 41% of the HNE at the second measurement point was explained by the HNE at t1 and that neither the control variables nor the actual usage data of the MuPDF reader explained further variance significantly.

**Table 3:** Regression analysis to predict the quality of families’ HNE at t2, by the HNE at t1, family and child characteristics and actual usage data of the MuPDF Reader

Model	Variables	β	*SE*	*t*	*p*	Adjusted R^2^
1	HNE1	0.64	0.03	18.397	0.001	0.413 **
2	HNE1	0.65	0.03	18.098	0.001	0.409
	Sex^a^	0.03	0.40	0.738	0.461	
	Age	0.02	0.00	0.556	0.578	
	Intelligence	−0.01	0.00	−0.177	0.860	
	STEM ^b^	−0.02	0.04	−0.477	0.633	
3	HNE1	0.65	0.03	18.074	0.001	0.410
	Sex^a^	0.03	0.04	0.798	0.425	
	Age	0.02	0.00	0.530	0.596	
	Intelligence	−0.003	0.00	−0.088	0.930	
	STEM ^b^	−0.02	0.04	−0.604	0.546	
	MuPDF	0.05	0.00	1.335	0.183	

*Note. N* = 495. β = standardized regression coefficients, ^a^ female = 1, male = 0, ^b^ Non-STEM = 0, STEM = 1; **p < 0.001. HNE1 = home numeracy environment at t1, STEM = parental STEM occupation

## Discussion

Different intervention approaches have been proposed in recent years for the support of children’s numeracy competencies (Lee & Choi, 2020; Niklas et al., 2020; Papadakis et al., 2018). Digitalization and digital interventions may support children’s development from the early years onwards (Berkowitz et al., 2015; Lee & Choi, 2020; Niklas et al., 2024; Papadakis et al., 2018), but at the same time, less attention has been paid to families’ home learning environment (see Eason et al., 2022; Niklas et al., 2021).

The present study evaluates a digital intervention approach with the aim to support children’s numeracy competencies as well as families’ HNE independent of family background characteristics (STEM vs. Non-STEM) in the early years by the means of learning apps and digital parent information. Our results indicate that preschool children’s numeracy competencies can be influenced positively by using high-quality learning apps. However, we did not find a positive impact of digital parent information on the quality of families’ HNE. Additionally, parents’ STEM occupation was not significantly associated with children’s numeracy and families’ HNE development between t1 and t2. Therefore, our results strengthen the assumption that digital interventions may appeal to and can support all families independent of their background.

### Supporting children’s numeracy competencies with a digital tablet-based intervention approach

Aligning with other studies (Berkowitz et al., 2015; Moyer-Packenham et al., 2016; Papadakis et al., 2018), our findings reveal that a tablet-based intervention can support the development of children’s early numeracy competencies. The usage of meaningful learning apps may lead to significant gains in children’s numeracy competencies, even after considering several control variables (Kim et al., 2021; Moyer-Packenham et al., 2016; Outhwaite et al., 2019; Papadakis et al., 2021). This result strengthens theories that emphasize app affordances such as deliberate practice or promoting active learning (Griffith et al., 2020; Hirsh-Pasek et al., 2015). In addition, we believe that the quality of our apps plays a decisive role (see Hirsh-Pasek et al., 2015), particularly given that total usage times were relatively low (about 40 min per week). However, this assumption needs to be investigated and analyzed more closely, since no consistent associations for the dosage of app-based interventions and the quality of apps for children’s development has been found yet (Kim et al., 2021). However, Berkowitz et al. (2015) showed that the quality of an app seems to be crucial and that the aspect of social interactivity plays a particularly important role. This aspect received less attention in our apps, and should be given greater focus in future app development (see also Wirth et al., 2024). We believe that more interactive apps would lead to greater long-term effects compared to children using a single app on their own. Another reason for the rather small effect size in our study could be, that our numeracy assessments were based on standardized tests rather than researcher-designed measures. As this aspect was reported as the most powerful moderator variable in a meta-analysis of Kim et al. (2021) on app-based learning it needs to be considered in future studies.

Our findings and findings of other studies underline the need for more research on the associations between apps developed based on educational intended frameworks such as the framework of Hirsh-Pasek et al. (2015), and Papadakis et al. (2020) and children´s competency development. Further, more app ratings and evaluations by children, parents, and experts (see Wirth et al., 2024) are needed to identify specific aspects of learning apps that are of particular educational value.

### Supporting families’ HNE by a digital tablet-based intervention approach

To date and to the best of our knowledge, little is known about digital interventions in the context of families’ HNE (see Niklas et al., 2021), despite recent research focusing on the quality of the HNE in analog and digital contexts (Lehrl et al., 2021, Niklas et al., 2016, Susperreguy et al., 2020b). Our findings demonstrate that the digital availability of parent information concerning children’s numeracy development via a PDF reader is not enough to change the HNE. We did not find a significant change in the quality of families’ HNE, even when comparing subsamples of parents who either used the information or did not use the information at all.

This finding stays in contrast with other mostly analog interventions that provided math-related information and increased families’ math engagement at home (Dulay et al., 2019; Niklas et al., 2016). Most of the studies (e.g., Niklas et al., 2016; Starkey & Klein, 2000) combined the provision of information with further activities for the parents, such as information evenings for the parents and recurring meetings on how to support children’s numeracy competencies at home. However, Cohrssen et al. (2023) also reported similar non-significant findings when using a messenger app as a tool to send informative short videos about children’s early development to parents to enhance the quality of the HNE in the families during the COVID-19 pandemic. However, a few studies evaluating parent support programs (e.g., EasyPeasy, Chancenreich), which did not focus specifically on mathematical interactions, but rather on general suggestions to support children’s development by enriching parent–child interactions at home through digital parent information, indicate a successful improvement in the quality of interactions, engagement and parents self-efficacy through the use of digital parenting apps (Anders et al., 2023; Jelley et al., 2016).

Consequently, important questions need to be posed: What is needed to support families’ HNE digitally, and how can families be encouraged within a digital intervention approach to support their children’s learning? One idea would be to create a specific app for parents containing all parent information instead of PDF files. Further, push notifications were not effective as children were the main users of the tablets in our study. Many parents never used the tablet and therefore never accessed our parent information, despite being informed about the content provided. Parents mostly viewed the tablet as a tool for their children rather than for themselves. As a result, they did not use the tablet or checked it regularly for parental information. Thus, the use of a parent app alone might have improved access to information while simultaneously enriching parent–child-interactions. In this context, it is also conceivable that push notifications could have been more effective, as reported in other evaluation studies of parent support programs using parent apps (York et al., 2019).

Reminding the parents via e-mail or other channels might have been more effective in the context of our study design, as stated by Ramani and Scalise (2020), who used various types of reminder methods (e-mail, text messages or paper slips every week) more frequently (once a week) to remind families about their card game intervention. However, more research on this topic is needed. It would be necessary to analyze parents’ needs and accessibility in order to pursue an intervention approach that is as sustainable as possible.

Interventions seem to be more efficient when both children and parents are approached simultaneously – such as when playing a digital game or a math-related app – while also receiving math-related information on how to interact with children. For example, Schaeffer et al. (2018) showed a positive intervention effect for children of parents with higher math anxiety when using an app together, which focused on children’s mathematical achievement and parents’ expectations. Further, Berkowitz et al. (2015) reported changes in parent–child interactions when using apps to engage in math-related topics. Zippert et al. (2019) documented an increase in parents’ and children’s math talks when playing a digital boardgame. However, both children and parents engaged in more math talk when parents were instructed in advance about math-related topics and how to interact with children concerning mathematics at home. Further, a combination of a digital and non-digital approach by combining mathematical apps and a board game may encourage families in interacting more intensively in the context of mathematics (see Griffith & Arnold, 2018). Accordingly, an approach, that is both parent- and child-centered may address parents more adequately and lead to greater quality in families’ HNE. In this context, apps that require parents and children to play together could be developed.

Apps thus represent an additional opportunity that can be integrated with everyday analog activities to support mathematical interactions, such as talking about numbers, playing games with numerical content, or doing math together. Furthermore, this could also be applied to the ECEC context, involving trained professionals and their guidance, as digitalization does not only impact the home learning environment but also the institutional learning context (Mues et al., 2024; Wirth et al., 2023). Ultimately, the overarching goal should be to combine all available learning opportunities and resources to support children’s development, tailored to their individual needs.

### Family characteristics to consider when investigating the effectiveness of digital intervention approaches on children’s numeracy competencies and families’ HNE

Our correlational analysis showed a significant association between parents’ STEM occupation, children’s numeracy development, and families’ HNE (see also Mues et al., 2021). Further, the between-subject effects demonstrate that children with parents with a STEM background showed higher starting numeracy values, but they did not show a significant difference between children with and without parents with a STEM background across the intervention period. Consequently, children profited from our digital math intervention, independent of their parents’ occupational background.

Similar results have been found for the HNE families provide for their children. Here, a positive association between families’ HNE and parents’ STEM background and the actual usage times spent by STEM-families was found in our correlational analysis. However, no significant differences in quality change of the HNE were found for families with STEM background compared to families without a STEM background. Still, it can be assumed that activities and interactions in families with a STEM background take place more frequently and are of a greater quality (Eason et al., 2022; Mues et al., 2021).

For example, Mues et al. (2021) showed such an association in a correlational analysis for parents with a learned STEM background, suggesting that the occupational background is reflected in the activities parents engage in with their children during numeracy-related activities at home. This assumption is further supported by findings indicating that parents are more likely to engage in and discuss their children’s early numeracy learning (Cheung et al., 2020). This is particularly visible, in a greater frequency of engagement in math language and both direct and indirect math teaching (Eason et al., 2022).

Additionally, this assumption aligns with the theory-based framework proposed by Eccles and Wigfield (2020), in their ‘parent-socialization model’, which describes the associations between direct and indirect parental characteristics and activities with children’s development. For instance, parental beliefs influence their actions and expectations, thereby indirectly affecting children’s competencies. Furthermore, the concept of a STEM capital (DeWitt et al., 2016) reinforces this argumentation. STEM capital encompasses various forms of cultural capital (such as science competencies, dispositions and knowledge about the transferability of science skills and qualifications), science-related behaviors and practices (e.g., exposure to science-related media, informal science experiences), and science-related social capital (e.g., parental science knowledge, conversations with others about science, encouragement to pursue science), all of which influence parental interactions with their children. These theories are supported by empirical findings demonstrating that parental involvement in children’s science or math learning, as well as engagement in STEM-related activities, is linked to parental beliefs and self-efficacy (Zucker et al., 2021).

Accordingly, these theories and empirical findings support our results and the assumption that parents with a STEM background engage more frequently and in higher-quality numeracy interactions. These families are more likely to have access to educational resources, exhibit science-related behaviors and practices, and possess science-related social capital. Therefore, parents with a STEM background may provide more structured, enriching, and frequent numeracy learning experiences for their children. However, clearly more research is needed on this topic. Further, these interactions support children’s numeracy development leading to higher numeracy scores for children from families with a STEM background (Mues et al., 2021). Consequently, family characteristics such as parental occupation (STEM vs. Non-STEM) and other factors such as parental beliefs, attitudes or the general SES are important predictors of children’s numeracy competencies, families’ HNE, and engagement in mathematical activities (e.g., Eason et al., 2022; Elliott and Bachman, 2018).

Future research needs to consider and investigate important family background characteristics when analyzing children’s numeracy development and families’ HNE while evaluating intervention approaches (Griffith & Arnold, 2018; Niklas et al., 2021). Further, we are still in need to investigate what accounts for variation in family math engagement, how digital technology may be associated with and impact the HNE and thus how it might support families’ HNE independently of family background characteristics.

## Limitations and future research

Several limitations of this study need to be considered when interpreting the findings. First, only the first two out of seven planned measurement points were used for our analysis. Accordingly, only immediate intervention effects were analyzed, and potential long-term effects still need to be investigated. Nevertheless, our first findings align with similar results of long-term interventions (e.g., Berkowitz et al., 2015).

Second, we used a unidimensional construct of children’s early numeracy competencies in our analyses. However, as stated by recent studies (e.g., Devlin et al., 2022) the construct of children’s numeracy competencies may not only be investigated by using a unidimensional approach, but may be examined as a multidimensional structure, so that various numeracy facets are analyzed separately. With such an approach, it would also be possible to assess which level (low, medium, high) of these competencies can be trained by app-based interventions.

Third, the descriptive data shows that parents did not use the provided information regularly, which weakened the intervention fidelity and is probably the reason we did not find any impact on the quality of the HNE.

Fourth, we used only self-reported data of parents to assess families’ HNE which might have led to biased and socially desirable answers (Zippert & Rittle-Johnson, 2020). Additionally, mostly mothers filled in our surveys as they were most often present during child assessments. However, mostly fathers were educated in STEM occupations. Consequently, results regarding math engagement at home and the use of digital devices and parent information might be mainly attributable to maternal responses. Considering both mothers’ and fathers’ answers might lead to a different understanding of parents’ engagement at home and their interactions about and with digital media (Hornburg et al., 2021; Mues et al., 2022). Consequently, new assessment tools that capture a broader concept, such as the Home Math Environment (Hornburg et al., 2021; Zippert & Rittle-Johnson, 2020), and/or include scales related to a digital home numeracy environment when working in the digital age (Alam & Dubé, 2023b) should be used and are needed. Considering aspects such as a general digital home learning environment (Lehrl et al., 2021) or digital home numeracy practices such as math app selection processes and parental perceptions of math app effectiveness (see Alam & Dubé, 2023b) could open up a new perspective when investigating the associations between families’ HNE and child outcomes. This approach could also provide further insights on families’ digital HNE and the interactions between parents and children.

Fifth, there are only a few categorization systems available that can be used as a guideline to classifiy parents’ STEM occupation. These systems categorize STEM occupations in very different ways and do not agree on a common definition. Therefore, it should be critically noted that not all occupations that could be considered as STEM-related or that promote mathematical skills are included in the category system we used.

Sixth, parental self-efficacy should be considered as an influencing factor when supporting the HNE digitally (Anders et al., 2023), as its association with the analog HNE has been demonstrated previously and is also recognized as an influencing factor in children’ numeracy competencies (Mues et al., 2022; Vasilyeva et al., 2018). Therefore, it would be interesting to look at the interaction of these aspects and a possible change in the intervention effect in future research.

Finally, researchers have identified negative associations between the (over)use of smartphones and tablets and various child developmental factors (e.g., poorer sleep, reduced physical activity, or cognitive difficulties; Bacil et al., 2024; Mallawaarachchi et al., 2022). However, Mallawaarachchi et al. (2022) also address methodological issues of such empirical studies (see also Griffith et al., 2020) and recommend to conduct more research, in particular studies designed as randomized controlled trials to facilitate “rigorous investigations of potential causality between distal and proximal influences on early childhood screen use and development” (Mallawaarachchi et al., 2022, p. 27). Consequently, it is necessary to further investigate advantages and disadvantages of the use of tablets and learning apps and other types of screen media to update recommendations for research and practice.

## Conclusions

Our results show that researcher-developed high-quality learning apps can support children’s numeracy development in early years independent of parental occupational STEM background. Therefore, such apps can be used in and recommended for practice regardless of the families children grow up in. However, our intervention approach was not sufficient to enhance the quality of families’ HNE. New tools and digital approaches are needed to involve parents in the intervention process and strengthen the HNE they provide. Such an intervention approach would guide and support parents in enriching their math engagement at home, for example through more frequent and higher-quality math talk (e.g., Besser et al., 2025), and would provide the basis for a joint parent–child exploration of STEM topics.

## Supplementary Information

Below is the link to the electronic supplementary material.

Supplementary file1 (DOCX 1237 KB)

Supplementary file2 (DOCX 81 KB)

## Data Availability

Data will be made available on request.
