# Supplementary material for: Supporting children’s numeracy competencies and families’ HNE: Exploring the role of apps and digital parent information in STEM vs. Non-STEM families
Source: Eur J Psychol Educ. 2025 Mar 21;40(2):53. doi: 10.1007/s10212-025-00953-7 (PMC11928358; doi:10.1007/s10212-025-00953-7)
Supplement: Supplementary file 1 — Supplementary file1 (DOCX 1237 KB) [file 10212_2025_953_MOESM1_ESM.docx]

**Table 1**

*Overview of learning apps used in the intervention*

| **App Name** | **Month** | **Description** | **Screenshot** | **Trained Skills** |
| --- | --- | --- | --- | --- |
| Memory (Numbers) | 1 | This app is based on the classic  analog memory game. All cards exist in pairs and start face down. Children tap the cards to flip them over and remember the numbers shown. The objective is to find and flip two matching cards consecutively. In the easier levels, number names are spoken aloud when the cards are tapped. | 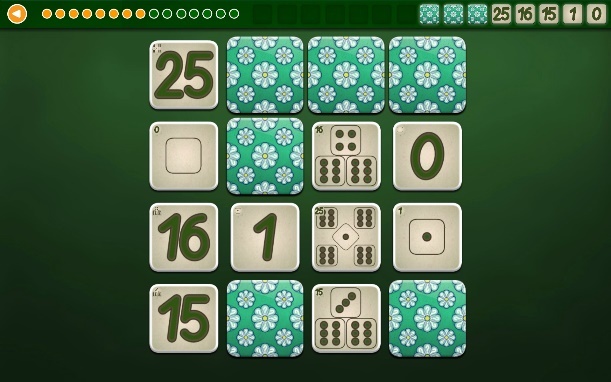 | Number symbol knowledge, knowledge of numeric representation |
| Number drawing | 1 | In this app, the goal is to trace the lines  with your finger by following the displayed patterns. Children follow an arrow on the screen and then trace whole number symbols. Each number is verbally provided as it is traced. | 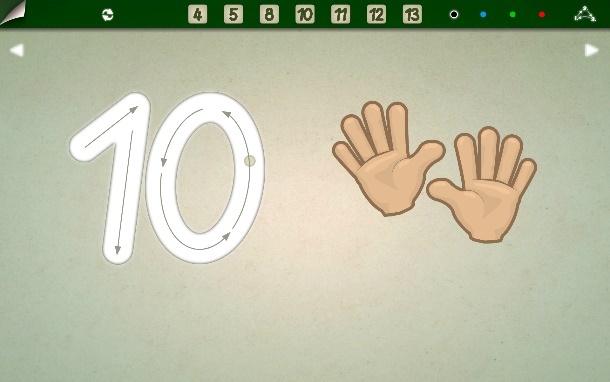 | Number symbol knowledge, knowledge of numeric representation |
| Painting with numbers | 1 | In this app, children color pictures.  Each area of the picture and each color is assigned a number. A legend shows which colors correspond to which numbered areas. When children tap, the number names are spoken aloud. | 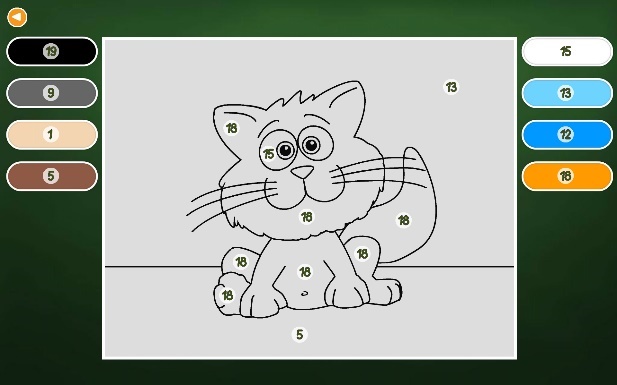 | Number symbol knowledge |
| Number sorting | 1 | In this app, children arrange a series of numbers in the correct sequence.  Their task is to correctly place the numbers, which are presented below in a random order and various forms of representation. The number names are provided verbally. | 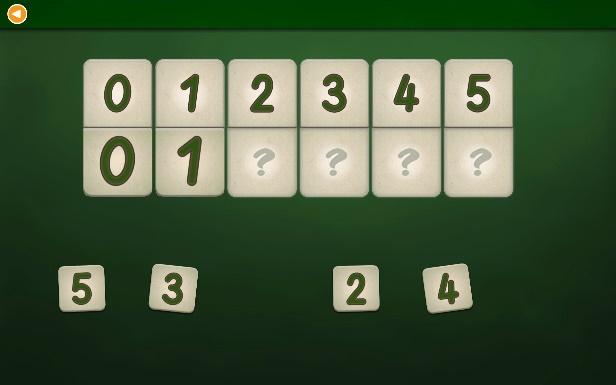 | Number symbol knowledge, Ordinal number knowledge |
| Build a number rocket | 1 | In this app, children build a number  rocket. On the right side of the screen, there is a rocket with empty spaces, while the left side displays various rocket parts with different numbers. To complete the rocket, children must arrange the parts in equal, ascending, or descending numerical order. Number names are provided verbally. | 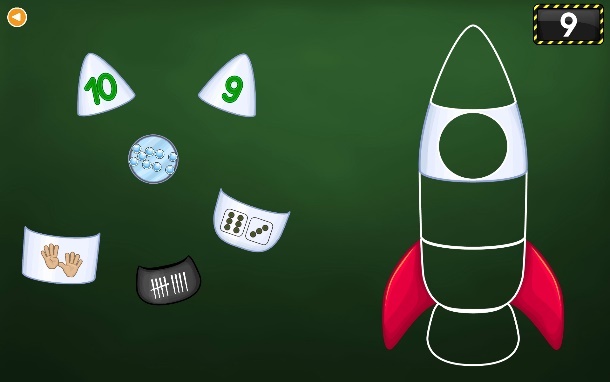 | Number symbol knowledge, knowledge of numeric representations, ordinal number knowledge |
| Collecting nuts | 2 | In this app, a squirrel follows a  predetermined path towards a destination. Children are informed of the number of different nuts the squirrel needs and need to tap the corresponding number of nuts accordingly. | 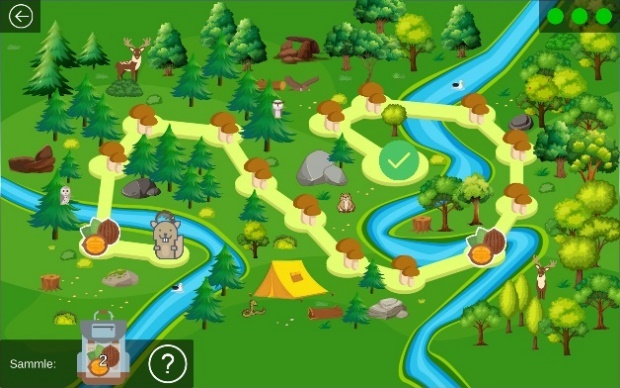 | Counting, inclusion and relations, number sequences forwards, cardinal number knowledge |
| Snakes & Ladders (Numbers) | 2 | This app is based on the traditional  Snakes & Ladders game. Both the board and the dice are labeled with numbers. It supports multiplayer mode, and the number names are spoken aloud during gameplay. | 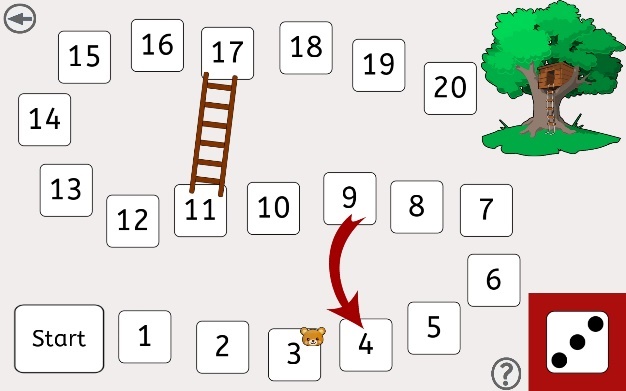 | Number symbol  knowledge, number  sequence forwards,  number sequence  backwards, number  predecessors and  successors |
| Tap it! Numbers | 2 | In this app, children are challenged to quickly select numbers by tapping them. They must find and tap a specified target number “x” while avoiding distractor numbers 1-3 that also appear regularly. Once tapped (or if not tapped fast enough), the numbers disappear. In a second phase of the game, children are tasked with counting. | 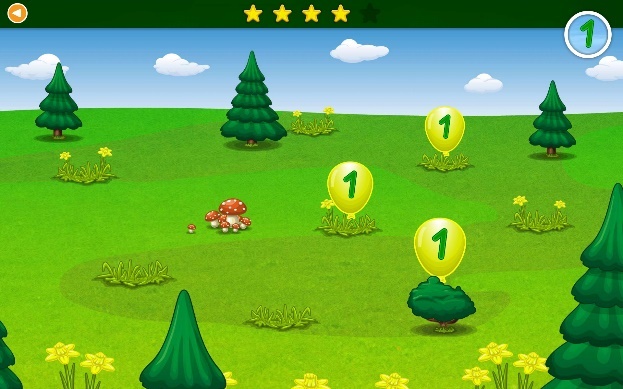 | Number symbol  knowledge, number  sequence forwards,  number sequence  backwards, number  predecessors and  successors |
| Mathemarmite | 2 | In this app, children create monsters by  using their counting skills to add the  correct number of specific ingredients  into a magic pot. The game focuses on  counting tasks, presenting numbers in various representations. | 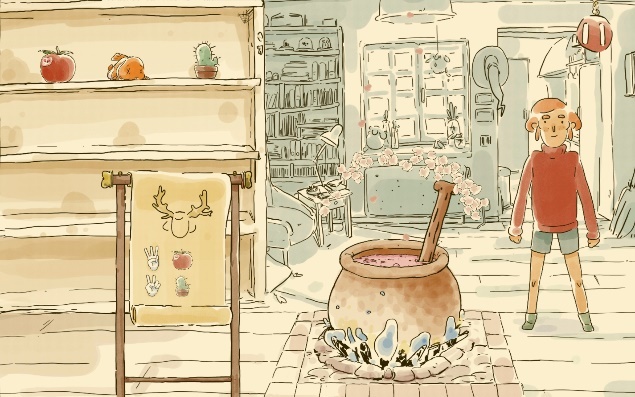 | Number symbol  knowledge, counting,  knowledge of numeric  representations |
| Measurement app | 3 | In this app, children encounter a ruler in the center of the screen. Objects and  animals of varying sizes appear alongside the ruler. Below the ruler, there is a number box where children are tasked with measuring the correct size and selecting the corresponding number card In more advanced levels, they are also required to replicate the size of the objects or animals with building blocks. | 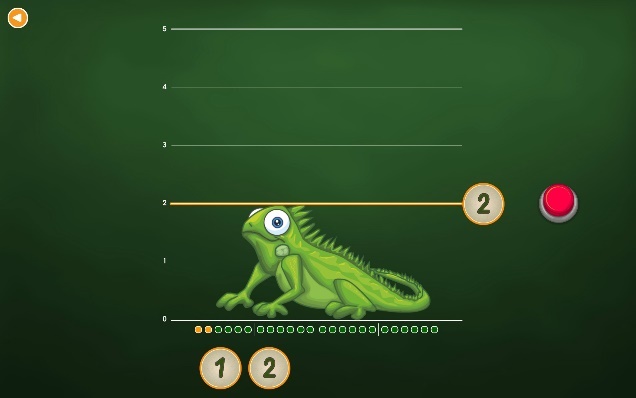 | Number symbol  knowledge, inclusion  and relations,  comparison and  measurement, easy  calculations |
| Finding pairs (Numbers) | 3 | In this app, children get the task of matching one out of two to four objects to a given number. All numbers are provided verbally. | 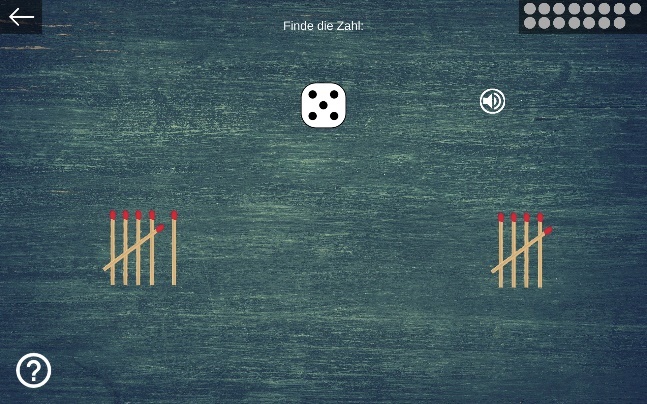 | Number symbol  knowledge;  Knowledge of numeric  representations |
| Count and compare | 3 | In this app, children practice counting animals and selecting the correct number card that corresponds to the counted amount. As they progress, they must identify the number of animals based on the given numeral, which is also spoken aloud. At advanced levels, they compare quantities (larger, smaller, equal). | 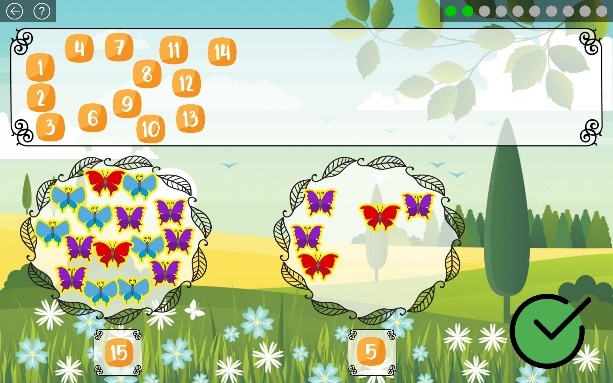 | Number symbol  knowledge, ordinal  number bars,  cardinality, inclusion  and relations,  counting, number  sequence forwards,  number sequence  backwards, |
| Counting the ballons | 4 | In this app, children count balloons and select the correct number of balloons. As they advance to more challenging levels, they engage in basic calculations involving the balloons*.* | 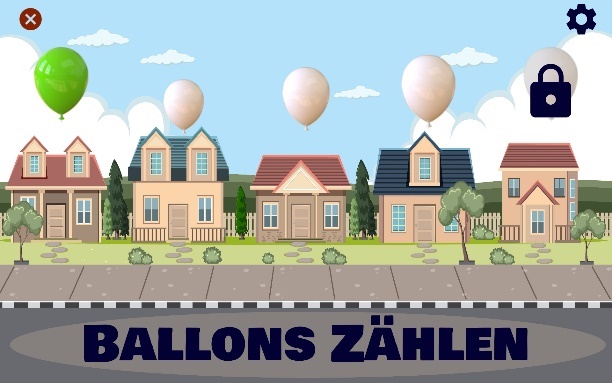 | Number symbol  knowledge, counting,  number sequence  forwards, number  sequence backwards,  addition & subtraction |
| Number-Domino | 4 | In this app, children arrange game pieces similar to a classic domino game and solve the puzzles correctly. The numbers on the dominoes vary in size and representation. | 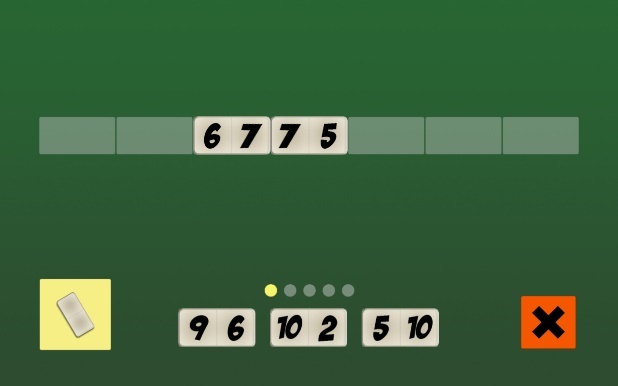 | Number symbol  knowledge, knowledge  of numeric  representations |
| Connect the number dots | 4 | In this app, children are asked to paint or complete a picture by connecting dots to form lines. The dots are marked with numbers indicating their sequence. As the levels progress, the numbers increase up to 20, and children are challenged to connect them in reverse order. | 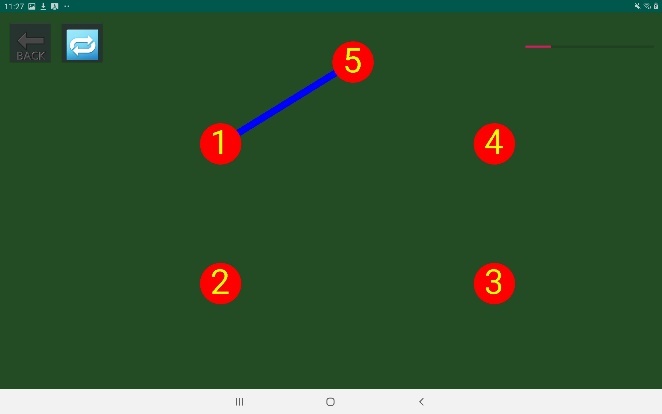 | Number symbol  knowledge, knowledge  of numeric  representations,  counting, number  sequence forwards,  number sequence  backwards |
| Learn the Clock | 5 | With this app, children learn to tell time. Initially, the app displays times using whole hours. As they progress, children identify times on the clock with smaller units. In more advanced stages, they engage in simple calculations involving time periods. | 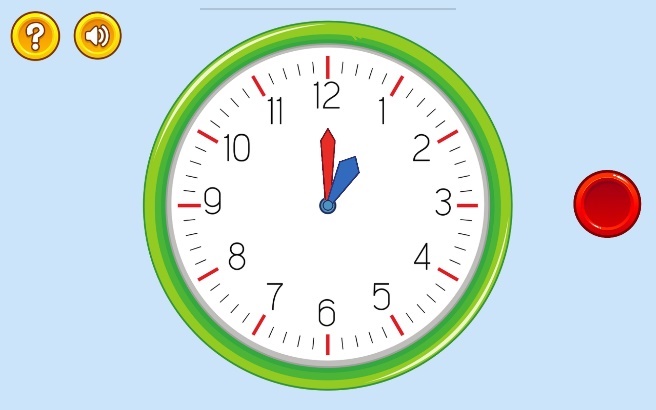 | Knowledge of numeric representation, cardinality, number division |
| Count and sort objects | 5 | In this app, children count various objects and match them to the corresponding number symbols on a box. | 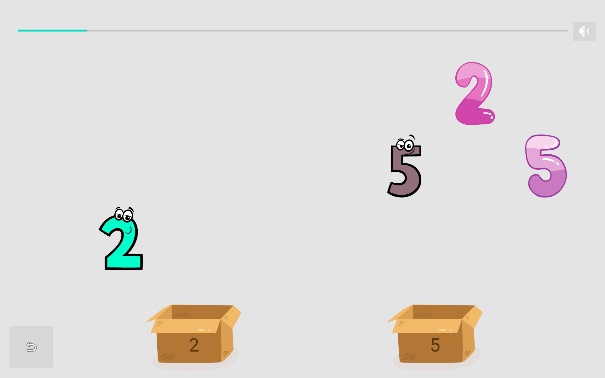 | Numbers, cardinality, number division, inclusion and relations, Number sequences forward, Number sequences backwards; Number predecessors and successors, Mathematical multiplication and subtraction |
| Categorize numbers | 5 | In this app, children find identical objects and arrange them in a row. Afterwards, they count the objects and match them with the correct number. | 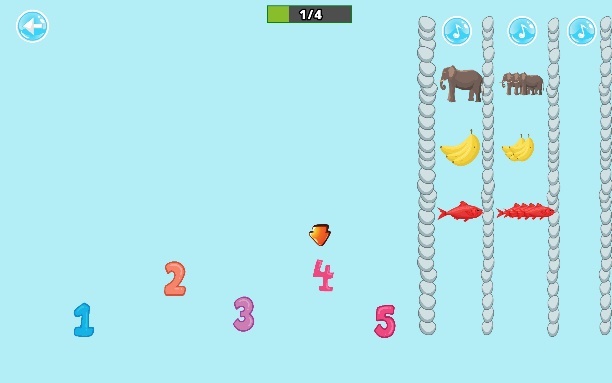 | Numbers, cardinality, number division, inclusion and relations |
