# Supplementary material for: Supporting children’s numeracy competencies and families’ HNE: Exploring the role of apps and digital parent information in STEM vs. Non-STEM families
Source: Eur J Psychol Educ. 2025 Mar 21;40(2):53. doi: 10.1007/s10212-025-00953-7 (PMC11928358; doi:10.1007/s10212-025-00953-7)
Supplement: Supplementary file 2 — Supplementary file2 (DOCX 81 KB) [file 10212_2025_953_MOESM2_ESM.docx]

|  |  | **Parent information** | **Tips** |
| --- | --- | --- | --- |
| Month 1 | Week1 | Mathematics in everyday life | Tip 1 Playing and math learning |
|  | Week 2 |  | Tip 2 Playing and math learning |
|  | Week 3 |  | Tip 3 Playing and math learning |
|  | Week 4 |  | Tip 4 Playing and math learning |
| Month 2 | Week 5 | Families as role models for numerical learning | Tip 5 Playing and math learning |
|  | Week 6 |  | Tip 6 Playing and math learning |
|  | Week 7 |  | Tip 7 Playing and math learning |
|  | Week 8 |  | Tip 8 Playing and math learning |
| Month 3 | Week 9 | Counting and comparing | Tip 9 Playing and math learning |
|  | Week 10 |  | Tip 10 Playing and math learning |
|  | Week 11 |  | Tip 11 Playing and math learning |
|  | Week 12 |  | Tip 12 Playing and math learning |
| Month 4 | Week 13 | Mathematical games | Tip 13 Playing and math learning |
|  | Week 14 |  | Tip 14 Playing and math learning |
|  | Week 15 |  | Tip 15 Playing and math learning |
|  | Week 16 |  | Tip 16 Playing and math learning |
|  | Week 17 |  | Tip 17 Playing and math learning |
| Month 5 | Week 18 | Measurement, forms, and teaching by the parents | Tip 18 Playing and math learning |
|  | Week 19 |  | Tip 19 Playing and math learning |
|  | Week 20 |  | Tip 20 Playing and math learning |
|  | Week 21 |  | Tip 21 Playing and math learning |

**Table 1**

*Overview of parent information and tips provided to the families***Example 1**

*Parent information on mathematics in everyday life*

Mathematics in everyday life

Dear parents,

here, we would like to provide you with some important information about the development and learning of your child. You may already know some of this and some information may be new to you! We invite you to read the following information (again and again) and to apply it with your child in everyday life.

Further, we will provide you with short tips and suggestions every week. You will also receive a longer text with additional information once a month in English and German. These texts will include brief, understandable descriptions of scientific work (see below at the end of the document). We hope you enjoy reading these documents!

*Introduction*

Many parents ask themselves: Why should I start learning with my child right now – won’t it be early enough for him/her to learn all this in school?

However, your child has been learning many new things every day since he/she was born (no, actually even earlier in the mother's womb).

Therefore, learning begins a long time before school starts. This is also the reason, why you cannot start learning together with your child too early - in a playful and fun way for you and your child of course.

And fun should always come first while learning with your kindergarten child. Your child only learns something new when he/she is motivated. If your child loses interest in an activity, it is often better to take a break.

In this case, it is better to interrupt the activity that you are doing together and continue later when your child has had a rest and is ready for more.

*Introduction*

Mathematics is a very important subject and formal maths learning for children starts at school. However, you can support your child to learn early mathematical knowledge before school. Such knowledge includes that your child knows the number names, learns to count correctly, is able to identify the number symbols, and is also able to compare, to add up or to divide small amounts.

In fact, studies (Butterworth, 2005, for details, please see below) show that we acquire first math skills very early. For instance, babies are already surprised when, one after the other, two dolls are moved behind a screen, and when then only one doll is visible when this screen is lifted. Further, even very young children are able to differentiate small amounts of up to three or four objects.

At the age of two to three years, many children start counting and with 3.5 years, many children can add or subtract the number 1 correctly to or from a given quantity. The early development of numeracy knowledge thus takes place in the context of the family environment long before school starts.

*Introduction*

It is important to note that the age-related information should only be regarded as a vague guideline. The development of mathematical competencies differs significantly from child to child.

A four-year-old child may still have big problems counting correctly to ten, while another child at the same age may easily be able to count correctly to 30. Such differences are found often at this age and are still quite normal!

But why is it important to practice early math skills with children? Isn’t it early enough for children to learn these competencies when they are at school? A longitudinal study (Krajewski & Schneider, 2009, for details, please see below), which analysed the development of children’s mathematical competencies provides a clear answer. The findings show that children who had acquired more mathematical knowledge in kindergarten are later also the children who do better in maths at school.

Consequently, it is worthwhile to support children’s early mathematical learning early on. Some good news for you: you can support such learning readily in everyday life! In our weekly tips, we provide you with some suggestions, how you may best support your child. You will receive this information weekly in a new document for you to read.

*Detailed information*

It is certainly not necessary for you as main caregiver of your child, to "train" your child and to play “math teacher”. However, you may support your child’s numeracy learning by offering opportunities for your child to get in contact with mathematics. Your child may develop an interest in mathematics only if he/she is in daily contact with mathematical content in everyday life they.

You should also observe your child, and when, for example, he/she counts or is engaged in activities with numbers, you should support their learning. How can you use everyday life for mathematical learning?

First, it helps to be aware of mathematical content that can be found almost everywhere around us. For instance, we encounter numbers in many facets in everyday life. We find numbers

- as numbers on sports jerseys,

- on price tags when shopping,

- as house, bus and train numbers,

- on watches,

- on the thermometer,

- when weighing and measuring objects,

- as a measure of speed (e.g., the speed of a car).

*Detailed information*

This list could easily be continued and shows the meaning of numbers and number symbols. Further, it is obvious that numbers are always found or used in our life. Consequently, you can easily learn math in everyday life.

Such learning is playful and you don't necessarily need expensive tools, learning programs or games. Mathematics surround us in our lives almost all the time. It doesn't matter whether you read the actual printed numbers on a watch, a price tag or a bus number, or whether you count amounts of things, or whether you think about how often we have to sleep until it is weekend again.

Consequently, it is also not necessary that you as parents need to plan, prepare and control your children's mathematical learning. Rather, you should simply try to put on “mathematical glasses”. This means that you should try to notice and use the mathematical aspects already existent in everyday situations and activities.

Your goal should be to identify mathematical content that exists in all of your child's activities (e.g. number symbols on toys or objects that can be counted).

*Detailed information*

In addition, it is also very important to correctly assess your child's mathematical skills. This means you should try to find out how advanced your child’s mathematical thinking is, what he/she already can do easily or for which tasks he/she still needs some support.

For instance, it will be of little use to teach your child the numbers between 10 and 20 or counting up from 20, if your child has not yet understood and mastered the numbers from 1 to 9 or counting up to 20. It is important to provide your child with tasks that fit to your child’s current mathematical knowledge.

However, the effort you put into these tasks is worthwhile: Studies (Niklas, Cohrssen & Tayler, 2016, for details see below) show that children have better early mathematical knowledge and also show greater gains in mathematical knowledge, when they come into contact with mathematics in everyday family life.

*Detailed information*

For instance, you should regularly count things with your child in the surroundings. Further, you may try to involve your child in cooking or paying. You may also explain how many degrees it is outside and where we can check the temperature, how fast you are driving and which numbers are on the road signs or on letterboxes and houses.

Simply use your everyday life and your surroundings! Next month, we will talk more about “numbers” and about your role as “mathematical role model” for your own children.

*Studies 1*

Brian Butterworth describes in a comprehensive and very interesting article the development of early math skills from birth. The following table shows some of the most important milestones on this page and the following. Please note that the age information should only be regarded as approximate indicator and that different children will master some of these abilities earlier or later.

Milestones in the development of mathematical skills (adapted from Butterworth, 2005, p. 12):

| **Children’s age** | **Milestones** |
| --- | --- |
| From day one | Can discriminate on the basis of small numerosities |
| From the age of 4 months | Can add and subtract one |
| About 1 year | Discriminates increasing from decreasing sequences of numerosities |
| 2 years | Begins to learn sequence of counting words |
| 2,5 years | Recognises that number words mean more than one |
|  |  |
| **Children’s age** | **Milestones** |
| 3 years | Counts out small numbers of objects |
| 3,5 years | Can add and subtract one with objects and number words |
| 4 years | Can use fingers to aid adding |
| 5 years | Can add small numbers without being able to count out sum |
| 6 years | Children understand, that the total number of things is independent of their order |
| 7 years | Children have memorized some arithmetical facts and they can retrieve them from memory |

As can be seen in the table, children develop important mathematical skills early on. They also learn important mathematical knowledge in the family context. Here, adults can support them without much effort.

*Studies 2*

In a longitudinal study with about 150 kindergarten children, Krajewski and Schneider (2009) analysed the development of mathematical skills in children. The same children with whom they worked in kindergarten were tested again later at the end of 1st and at the end of 4th grade.

In kindergarten, the children were asked to count forwards and backwards, name number symbols, recognize which number comes before or after another number, and do simple counting and arithmetic tasks. All of these abilities are so-called mathematical precursors, which are good predictors of later mathematical skills. The same children then participated in standardized mathematics tests at school, which were based directly on the curriculum and thus represented the mathematical school performance very well.

What kind of relationship did the researchers find between children's early mathematical abilities and later mathematical performance in school?

In fact, mathematical precursors were able to predict later mathematical performance very well, even when the social status of the parents, the intelligence of the children and their memory abilities were taken into account. These findings indicate that children who were already better at counting in kindergarten, who were able to identify more number symbols and who were better at counting and doing simple calculations, were also the children who had fewer problems with school mathematics. Conversely, the children with the weakest mathematical performance at school already had significantly poorer mathematical precursors in kindergarten compared to their peers.

*Studies 3*

In an Australian study conducted by Niklas, Cohrssen and Tayler (2016), parents received relevant information about the importance of the family for early mathematical learning during a parental evening in their children's kindergarten. Here, the parents were told, for example, that they should use everyday opportunities to name numbers, count things, play games with mathematical content and compare amounts ("little or a lot", "who has more, who has less?"). Further, parents were invited to show their children how important mathematic is in everyday life and that we use mathematics all the time, e.g. when we pay, cook, take the time or count cutlery.

The researchers also met the parents for a short game session with their child. A simple dice game was played, in which the parents and children rolled the dice, had to count the correct number of tokens and thus practiced counting and comparing of amounts.

These simple measures led to mathematical interactions being carried out more frequently and more consciously in everyday family life. Consequently, the quality of the home numeracy environment was improved. This also had the effect that the children of parents who had participated in the intervention showed a greater gain in their mathematical precursor abilities compared to children whose parents had not participated.

This study thus shows that it is well worth using everyday life for mathematical learning.

**Example 2**

*Tip on mathematics in everyday life*

*Tips 3*

While changing clothes, "mathematical interactions" are readily introduced: "Which clothes are bigger or

smaller?", "Where are more buttons?”, “How many buttons are on my blouse/shirt?", "How many clothes do we

wear?”, “How long does it take to change clothes for you and for me? Let’s count together”, etc.

*References*

*3*

Butterworth, B. (2005). The development of arithmetical abilities. Journal of Child Psychology and Psychiatry, 46(1), 3–18. <https://doi.org/10.1111/j.1469-7610.2004.00374.x>

Niklas, F., Cohrssen, C., & Tayler, C. (2016). Improving Preschoolers’ Numerical Abilities by Enhancing the Home Numeracy Environment. Early Education and Development, 27(3), 372–383. https://doi.org/10.1080/10409289.2015.1076676

Krajewski, K., & Schneider, W. (2009). Early development of quantity to number-word linkage as a precursor of mathematical school achievement and mathematical difficulties: Findings from a four-year longitudinal study. Learning and Instruction, 19(6), 513–526. https://doi.org/10.1016/j.learninstruc.2008.10.002
